# Identifying Quantitative Trait Loci and Candidate Genes Conferring Resistance to *Soybean Mosaic Virus* SC7 by Quantitative Trait Loci-Sequencing in Soybean

**DOI:** 10.3389/fpls.2022.843633

**Published:** 2022-02-28

**Authors:** Yong Zhang, Jiling Song, Lei Wang, Mengping Yang, Kaifeng Hu, Weiwei Li, Xuhong Sun, Hong Xue, Quanzhong Dong, Mingming Zhang, Shubao Lou, Xingyong Yang, Hao Du, Yongli Li, Lidong Dong, Zhijun Che, Qun Cheng

**Affiliations:** ^1^Keshan Branch of Heilongjiang Academy of Agricultural Sciences, Qiqihar, China; ^2^Innovative Center of Molecular Genetics and Evolution, School of Life Sciences, Guangzhou University, Guangzhou, China; ^3^School of Agriculture, Ningxia University, Yinchuan, China

**Keywords:** soybean, QTL, *Soybean mosaic virus*, SC7 strain, QTL-seq, MATE transporter

## Abstract

*Soybean mosaic virus* (SMV) is detrimental to soybean (*Glycine max*) breeding, seed quality, and yield worldwide. Improving the basic resistance of host plants is the most effective and economical method to reduce damage from SMV. Therefore, it is necessary to identify and clone novel SMV resistance genes. Here, we report the characterization of two soybean cultivars, DN50 and XQD, with different levels of resistance to SMV. Compared with XQD, DN50 exhibits enhanced resistance to the SMV strain SC7. By combining bulked-segregant analysis (BSA)-seq and fine-mapping, we identified a novel resistance locus, *R_*SMV*_-11*, spanning an approximately 207-kb region on chromosome 11 and containing 25 annotated genes in the reference Williams 82 genome. Of these genes, we identified eleven with non-synonymous single-nucleotide polymorphisms (SNPs) or insertion-deletion mutations (InDels) in their coding regions between two parents. One gene, *GmMATE68* (*Glyma.11G028900*), harbored a frameshift mutation. *GmMATE68* encodes a multidrug and toxic compound extrusion (MATE) transporter that is expressed in all soybean tissues and is induced by SC7. Given that MATE transporter families have been reported to be linked with plant disease resistance, we suggest that *GmMATE68* is responsible for SC7 resistance in DN50. Our results reveal a novel SMV-resistance locus, improving understanding of the genetics of soybean disease resistance and providing a potential new tool for marker-assisted selection breeding in soybean.

## Introduction

Soybean [*Glycine max* (L.) Merr.] is one of the most important sources of plant protein and vegetable oil, providing more than one-quarter of the world’s protein for food and animal feed (Hoeck et al., 2003; Boerma and Specht, 2004). Breeding soybean varieties with both high seed quality and high yield remains an important goal of breeders. Both traits can be strongly influenced by pathogen attack. For example, soybean mosaic virus (SMV), a single-stranded, positive-sense RNA virus of the genus *Potyvirus*, causes losses in soybean yields and seed quality worldwide (Ross, 1977; Hajimorad et al., 2018). Combined infection of soybean with SMV and bean pod mottle virus (BPMV) (genus comovirus, family comoviridae) further reduces yield, by up to 85% (Ross, 1968). Thus, SMV resistance is a critical trait in soybean. However, a series of *Rsv* loci have been reported in soybean accessions and introduced into commercial varieties: *Resistance to SMV1* (*Rsv1*), *Rsv3*, *Rsv4*, and *Rsv5* (Kiihl and Hartwig, 1979; Buss et al., 1997; Hayes et al., 2000; Klepadlo et al., 2017). *Rsv1*, *Rsv3*, and *Rsv5* are SMV strain specific and are presumed to encode NB-LRR proteins (Cho and Goodman, 1979; Hajimorad and Hill, 2001; Suh et al., 2011; Klepadlo et al., 2017). *Rsv1* resistance alleles have been found in the cultivars Ogden, York, Marshall, Kwanggyo, Raiden, Suweon97, PI 486355, PI 507389, and FT-10 (Moon et al., 2009; Tucker et al., 2009), and confer resistance to SMV G1–G3 strains (Chen et al., 1991). In contrast, *Rsv3* confers resistance to SMV G5–G7strains (Gunduz et al., 2002). Interestingly, *Rsv4* confers broad-spectrum SMV resistance through an atypical mechanism that delays viral proliferation (Gunduz et al., 2004). The *Rsv4* locus was first reported in 1995, and its molecular characterization has recently been achieved: it encodes an RNase H family protein with double-stranded (ds) RNA-degrading activity that enters the viral replication compartment and degrades viral dsRNA (Ishibashi et al., 2019).

Over the past several decades, extensive efforts have been made to identify SMV resistance loci and genes in soybean. In China, 22 SMV strains (SC1–SC22) have been identified (Wang et al., 2003, 2011; Yan et al., 2015), along with several resistance loci. The resistance genes *Rsa*, *Rn1*, *Rn3*, *Rsc7*, *Rsc8*, *Rsc9*, *Rsc13*, and SC18-resistance gene derived from the cultivar Kefeng 1 were mapped to chromosome 2 (Wang et al., 2003; Cai et al., 2014; Yan et al., 2015; Li et al., 2015). The resistance gene *Rsc15*, derived from RN-9, was mapped to chromosome 6 (Yang and Gai, 2011). The genes *Rsc3*, *Rsc11*, *Rsc12*, and *Rsc14* derived from Qihuang 1 were mapped to chromosome 13 (Li et al., 2010; Ma et al., 2011; Cai et al., 2014; Zheng et al., 2014). Finally, *Rsc4* derived from Dabaima was mapped to chromosome 14 (Wang et al., 2011).

Despite the identification of several SMV resistance loci and genes over the past several decades, breeding for SMV resistance remains challenging, requiring many time-consuming and laborious artificial inoculation experiments in the greenhouse or field. Bulked-segregant analysis (BSA) is an effective method to identify DNA markers closely linked to the candidate gene for an extreme phenotype (Michelmore et al., 1991).

In this paper, we report a novel QTL for SC7 resistance, named *R_*SMV*_-11* (Resistance locus to SMV on chromosome 11). This QTL was identified by whole-genome resequencing of two DNA bulks of progeny showing extreme phenotypic values. Through combined fine-mapping and qRT-PCR analysis, we isolated five possible genes associated with *R_*SMV*_-11*. Our data proves important information for use in marker-assisted selection in soybean resistance breeding.

## Materials and Methods

### Plant Materials, Virus Inoculation, and Primer Design

The SC7-resistant soybean cultivar DN50 (Dongnong 50) used in this study was obtained from Northeast Agricultural University, Harbin. The SC7-susceptible soybean cultivar XQD (Xiaoqingdou) was obtained from the KeShan Branch of HeiLongJiang Academy of Agricultural Sciences, KeShan.

For SC7 inoculation, the seeds of DN50, XQD and near-isogenic inbred lines (NILs) of *R_*SMV*_-11* were planted in a greenhouse with a 16 h light/8 h dark photoperiod and maintained at 25^°^C with 70% relative humidity. Soybean plants were inoculated with SMV strain SC7 following the methods described by Che et al. (2020). The control leaves were carried out with equivalent amounts of 0.01 M sodium phosphate buffer (PH 7.2–7.4). Foliar symptoms were monitored every three days after inoculation.

Primers were designed online using Primer 5 based on the Williams 82 reference genome. All primers used for fine-mapping and qRT-PCR assays for candidate genes are listed in Supplementary Table 1.

### Genomic DNA and Total RNA Extraction

A single young leaf was collected from each plant at the V2 stage (one fully expanded trifoliate). Genomic DNA was extracted using the cetyltrimethylammonium bromide (CTAB) method (Saghaimaroof et al., 1984). Total RNA was isolated from 1 g soybean leaves using TRIzol (Invitrogen, Shanghai, China) according to the manufacturer’s protocol. The extracted DNA and RNA were quantified using a NanoDrop 2000C ultra-micro spectrophotometer (Sunnyvale, CA, United States) and 1.5% agarose gel electrophoresis.

### Segregation Population

A cross was generated between the resistant cultivar DN50 and the susceptible XQD. After self-pollination of F_1_ plants, 355 F_2_ seeds were harvested. All individual F_2_ plants were grown in a field in KeShan, China, under natural conditions and a bi-parental F_6_ recombinant inbred lines (RIL) population was developed by single-seed descent. The F_6_ RILs were planted in the field in KeShan, China, in 2018 for mapping of the *R_*SMV*_-11* locus.

### Bulking, BSA-Seq Analysis, and Genetic Mapping

Thirty highly resistant and 30 highly susceptible F_6_ individuals were screened to generate the R- and S-bulks, respectively. DNA samples of the parental lines and the two bulks were subjected to whole-genome resequencing using the Illumina HiSeq X Ten platform, followed by standard paired-end 150-bp sequencing library construction. Primer sequences of the markers for mapping are listed in Supplementary Table 1. For fine-mapping, 11 markers between positions 1,632,020 and 4,642,090 were identified. Six recombinants were identified in the fine-mapping population using 11 markers, and the SC7 resistance phenotype of their progeny was evaluated to delimit the genomic interval containing *R_*smv*_-11*. The genotypes of the *R_*smv*_-11* allele were analyzed by tagging marker M24 or M28.

### Quantitative Real-Time PCR

cDNA was synthesized from total RNA using an Oligo (dT) 18 primer and PrimeScript 1st strand cDNA Synthesis Kit (Takara, Dalian, China). qRT-PCR analysis was performed to determine the transcript abundance of candidate genes using LightCycler 480 SYBR Green I Master (Roche, Mannheim, Germany) in a LightCycler 480 system (Roche, Mannheim, Germany). The soybean housekeeping gene *Tubulin* was used as the internal control. The relative transcript level of the target gene was calculated using the 2^–ΔΔCt^ method. Three biological replicates with three technical replicates each were performed. The expression data of candidate genes in different soybean tissues (leaf, stem, root, flower, seed, pod, and nodule) were obtained from the RNA-seq database (Machado et al., 2020). Primers used are listed in Supplementary Table 1.

## Results

### Parental Soybean Lines Exhibit Variation in Resistance to the SC7 Viral Strain

**FIGURE 1:**
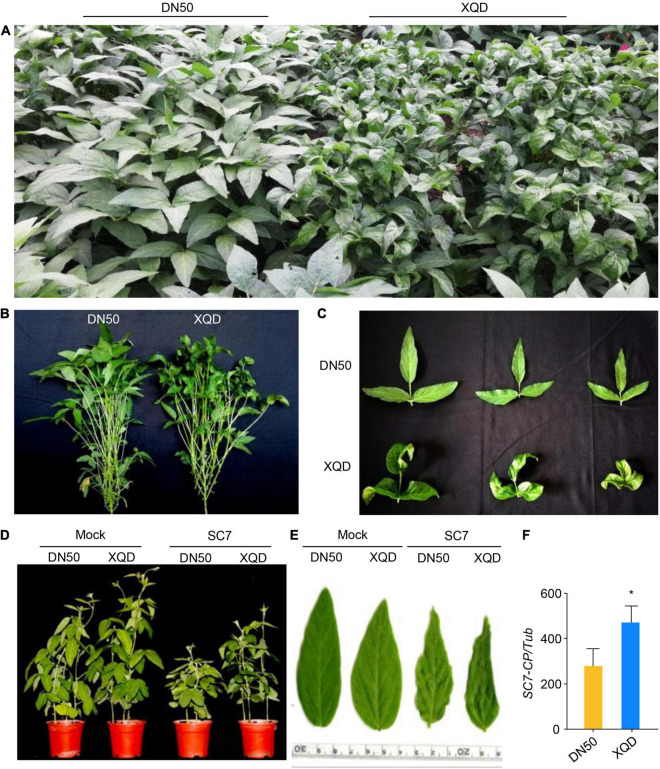
Phenotypes of two soybean cultivars, DN50 and XQD, inoculated with soybean mosaic virus strain SC7. **(A,B)** Phenotype of DN50 and XQD inoculated with SMV in the field. **(C)** Leaf phenotypes. DN50 shows partial resistance, whereas XQD is susceptible. **(D)** Phenotype of DN50 and XQD inoculated with SC7 in greenhouse. **(E)** Leaf phenotype. **(F)** Accumulation of SC7 in DN50 and XQD SC7-inoculated leaves. The amplification of soybean TUB (*GmTubulin*) was used as an internal control to normalize all data. Data from three biological replicates are shown, each with three technical replicates. Statistically significant differences were determined using Student’s *t*-test (*P < 0.05). Error bars indicate standard error of the mean.

To assess the variation in disease resistance between DN50 and XQD, we identified the phenotype of DN50 and XQD. The result domesticated soybean varieties DN50 and XQD, display clear differences in resistance to the SC7 viral strain in the field (Figures 1A–C). Under natural conditions, SC7 caused symptoms in up to 90% of leaves in XQD, but did not induce any symptoms in the leaves of DN50. Moreover, in the greenhouse, SC7-treated XQD exhibited enhanced rugosity, curling, and chlorosis symptoms compared with DN50 (Figures 1D,E). We also analyzed the relative accumulation of SC7 in top non-inoculated leaves at 21 days post inoculation (dpi) in the greenhouse. The accumulation of viral *CP* gene in DN50 was significantly lower than that in XQD (Figure 1F). Together, these results suggested that DN50 is more resistant than XQD to SC7.

### Mapping *R_*SMV*_-11*, the QTL Controlling Partial Resistance to SC7

**FIGURE 2:**
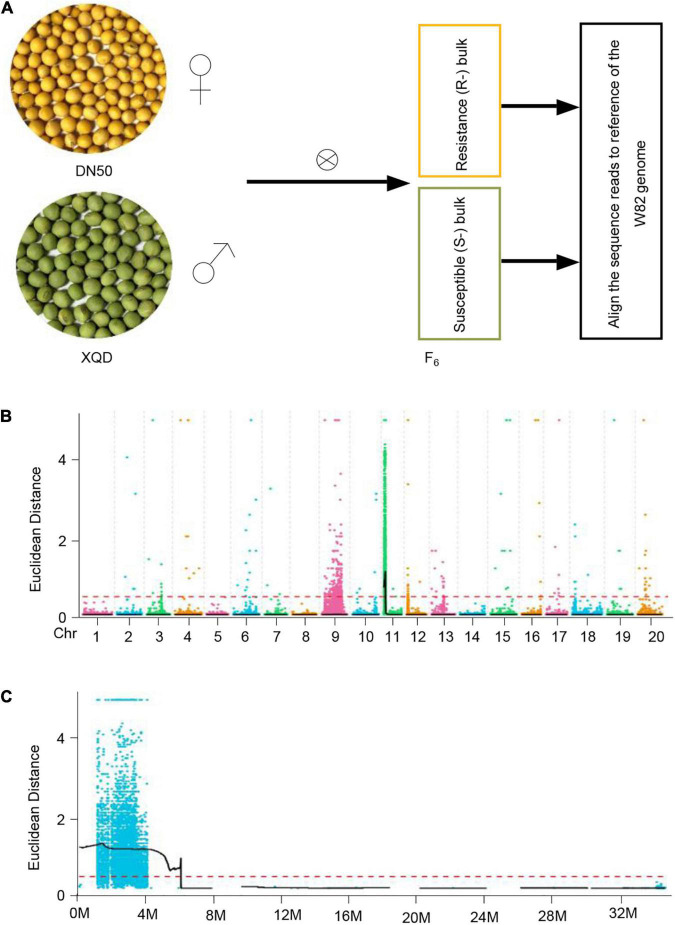
A simplified scheme of BSA-seq as applied to soybean. **(A)** Morphological difference in seed size between DN50 and XQD accessions. Two cultivars with different phenotypes are crossed to generate F6 progeny segregating for the trait value. Multiple progeny exhibiting resistance and susceptibility to SC7 are selected, and their DNA is bulked to produce “Resistant” and “Susceptible” bulks, respectively. **(B)** Examples of Euclidean distance (ED)-associated values on chromosomes. The color points represent the ED value of each single-nucleotide polymorphism (SNP) locus. The black line is the fitted ED value, and the red dotted line represents the significantly associated threshold. Higher ED values indicate stronger correlations. **(C)** The distribution of Euclidean distance (ED)-associated values on chromosome 11.

To understand the molecular basis of SC7 resistance, we aimed to detect genetic differences between SC7-resistant and -susceptible plants. To this end, we first generated an F_2_ population by crossing DN50 and XQD (Figure 2A). The F_2_ population by four rounds of self-fertilization generated an F_6_ population (Figure 2A). We selected two groups of F_6_ plants reflecting segregation of the SC7 resistance trait (phenotype of F_6_ populations revealed a 3:1 ratio of the resistance and susceptible to SC7): one group of 30 individuals showing resistance to SC7 and a second group containing 30 individuals susceptible to SC7 (Figure 2A).

Next, we sequenced the two bulked DNAs (R- and S-bulks), along with single DNA samples of the two parents (DN50 and XQD), on the Illumina HiSeq X Ten platform and then resequenced the genomes of the two parents at 30× coverage each and the two bulked DNAs at 20× coverage each (∼100 Gb data). After filtering, a total of 2,005,612 bi-allelic single-nucleotide polymorphisms (SNPs) and a total of 432,197 bi-allelic short insertions and deletions (InDels) were identified in the two parents, and 113,258 bi-allelic SNPs and 46,172 bi-allelic InDels were identified in the two bulked DNAs. We then aligned the resequencing data of the two bulked sample groups (R- and S-bulks) to the Williams 82 reference genome (*Glycine max* Wm82.a2.v2; Schmutz et al., 2010).

Using the genotype data from the parents and the two sample groups (R and S), we used the Euclidean distance (ED) algorithm to locate the SC7 resistance QTL locus. Only one QTL at the beginning of chromosome 11 was significantly associated with resistance to SC7; this QTL was named *R_*SMV*_-11* (Figures 2B,C). ED value analysis revealed that a physical region of 0.944 kb–3.418 Mb on chromosome 11 was possibly linked to SC7 resistance.

### Positional Cloning of the *R*_*SMV*_-*11* Locus

**FIGURE 3:**
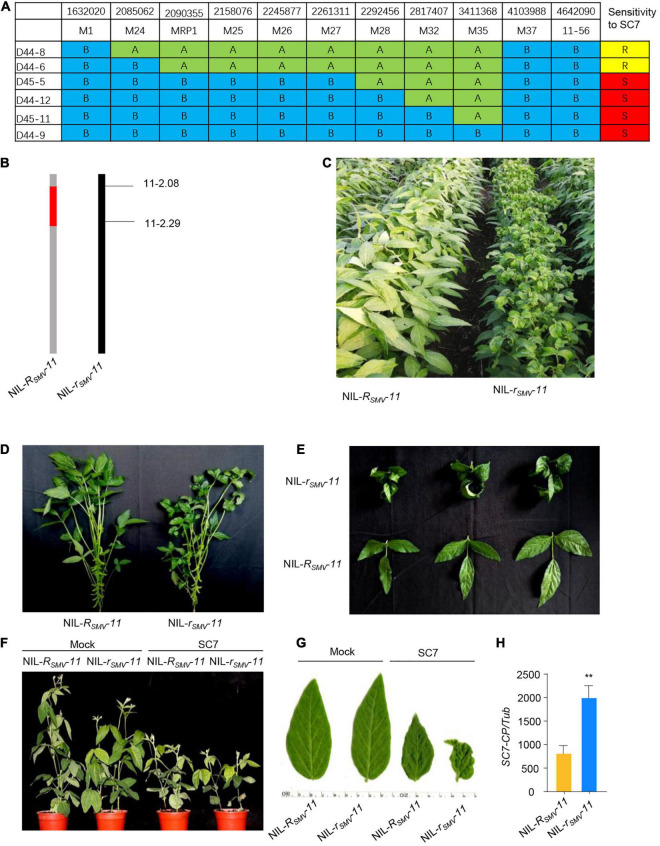
Fine-mapping of *R_*smv*_-11*. **(A)** Characterization of key recombinants from the F_6_ segregated population in the immediate vicinity of the *R_*smv*_-11* locus (*n* = 36 plants). “A”, *R_*smv*_-11* homozygous; “B”, *r_*smv*_-11* homozygous; “H”, heterozygous. “R”, Resistant to SC7; “S”, susceptible to SC7. **(B)** Chromosome maps of NIL-*R_*smv*_-11* and NIL-*r_*smv*_-11*. Red rectangles indicate the donor segment containing the *R_*smv*_-11* locus. **(C,D)** Phenotype of NIL-*R_*smv*_-11* and NIL-*r_*smv*_-11* inoculated with SC7 in the field. **(E)** Leaf phenotype. **(F)** Phenotype of NIL-*R_*smv*_-11* and NIL-*r_*smv*_-11* inoculated with SC7 in the greenhouse for 2 weeks. **(G)** Leaf phenotype. **(H)** Accumulation of *SC7* in leaves of NIL-*R_*smv*_-11* and NIL-*r_*smv*_-11* treated with SC7. The amplification of soybean *TUB* (*GmTubulin*) gene was used as an internal control to normalize all data. Data from three biological replicates are shown, each with three technical replicates. Statistically significant differences were determined using Student’s *t*-test (*P < 0.05). Error bars indicate standard error of the mean.

To more precisely map the *R*_*SMV*_-*11* gene within the previously identified candidate regions, we used the F_6_ RIL population. We localized *R*_*SMV*_-*11* to a 207-kb region between markers M24 and M28 (Figure 3A), adjacent to the *R*_*SMV*_-*11* region mapped by BSA-seq.

To substantiate the relationship between the *R*_*SMV*_-*11* QTL and SC7 resistance, we compared the phenotypes of two F_6_ near-isogenic lines (NILs) carrying either the functional *R*_*SMV*_-*11* allele (NIL-*R*_*SMV*_-*11*) or the non-functional *r*_*SMV*_-*11* allele (NIL-*r*_*SMV*_-*11*) (Figure 3B) in both the field and the greenhouse. The results showed that NIL-*R*_*SMV*_-*11* had greater resistance to SC7 in the field than NIL-*r*_*SMV*_-*11* (Figures 3C–E). Consistent with the field phenotypes, NIL-*r*_*SMV*_-*11* exhibited enhanced curling symptoms and chlorosis compared with NIL- *R*_*SMV*_-*11* in the greenhouse (Figures 3F,G). We also analyzed the relative biomass of SC7 in infected NIL-*R*_*SMV*_-*11* and NIL-*r*_*SMV*_-*11* soybean leaves at 21 days post infection (dpi) with SC7 in the greenhouse. The biomass of SC7 in NIL-*R*_*SMV*_-*11* was significantly lower than in NIL-*r*_*SMV*_-*11* (Figure 3H). These data confirm that the *R*_*smv*_-*11* allele could greatly enhance resistance to SC7, and that the *R*_*smv*_-*11* gene was in this candidate region.

### Identification of Candidate Genes in the Delimited Region

**FIGURE 4:**
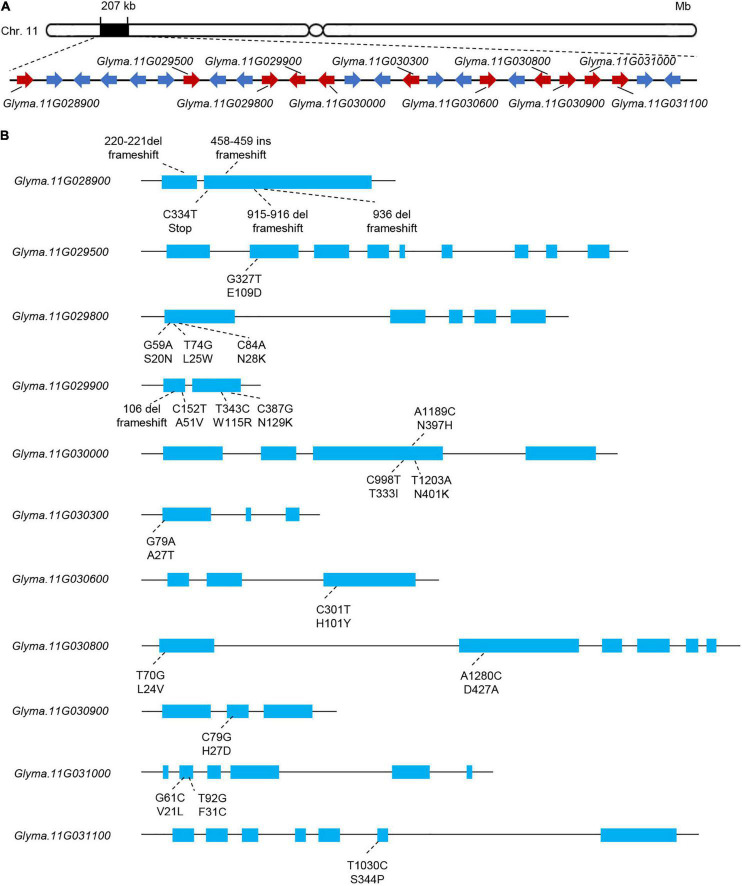
Polymorphism analysis of the candidate genes in the 207-kb region of interest. **(A)** Gene structure of *R_*smv*_-11* showing the location of the candidate genes. Red represents genes with different sequences between parents. **(B)** Gene structure and variation information of candidate genes with different sequences between parents.

In the 207-kb candidate mapping region, we found 25 genes (Figure 4A and Supplementary Table 2). We identified SNPs and InDels of all 25 genes in the two parents, and SNPs or InDels in 11 genes resulted in non-synonymous amino acid substitutions or frameshift mutation in the deduced protein sequences (Figure 4B). Of these 11 genes, only two (*Glyma.11G028900* and *Glyma.11G029900*) harbored InDels predicted to cause frameshifts (Figure 4B and Supplementary Table 3).

### Expression Analysis of Candidate Genes in Soybean Tissues

**FIGURE 5:**
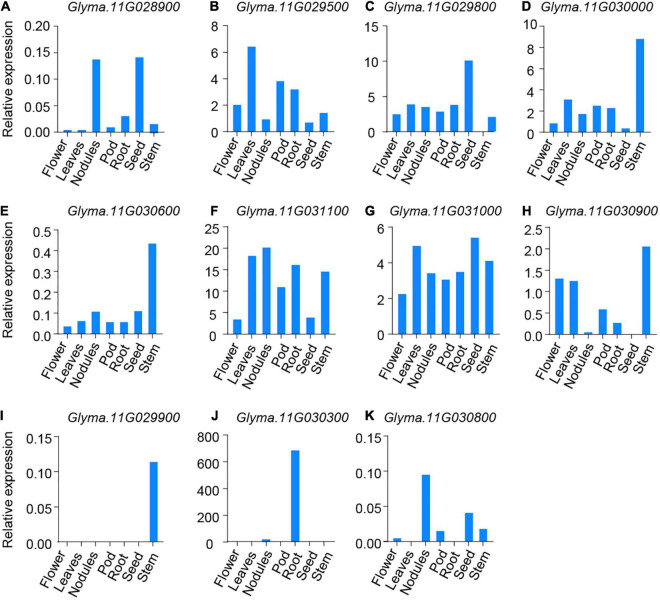
Expression of eleven candidate genes in different soybean tissues. **(A–K)** The expression of *Glyma.11G028900*, *Glyma.11G029500*, *Glyma.11G029800*, *Glyma.11G030000*, *Glyma.11G030600*, *Glyma.11G031100*, *Glyma.11G031000*, *Glyma.11G030900*, *Glyma.11G029900*, *Glyma.11G030300*, and *Glyma.11G030800* in different soybean tissues (flower, leaves, pod, stem, nodule, seed, and root) obtained from an RNA-seq database. Values are mean ± s.e.m. (*n* = 3 biologically independent samples).

To further screen candidate genes, we searched an RNA-seq database and retrieved the expression data for 11 candidate genes with DNA sequences differing between DN50 and XQD. We analyzed data for seven tissues: flower, leaves, pod, stem, nodules, seed, and root (Machado et al., 2020). Seven genes were constitutively expressed in all tissues: *Glyma.11G028900*, *Glyma.11G029500*, *Glyma.11G029800*, *Glyma.11G030000*, *Glyma.11G030600*, *Glyma.11G031100*, and *Glyma.11G031000* (Figures 5A–G). *Glyma.11G030900* was highly expressed in all tissues except the seeds (Figure 5H). The other three genes displayed low transcript abundance in most tissues; however, *Glyma.11G029900* showed high expression in the stem, *Glyma.11G030300* was highly expressed in roots, and *Glyma.11G030800* was abundant in nodules and seeds (Figures 5I–K). Given that *Glyma.11G029900*, *Glyma.11G030300*, and *Glyma.11G030800* were barely expressed in leaves, we excluded these three genes from this candidate region and subsequent analyses.

### Expression Profiling to Identify Resistance Genes From the Candidates

**FIGURE 6:**
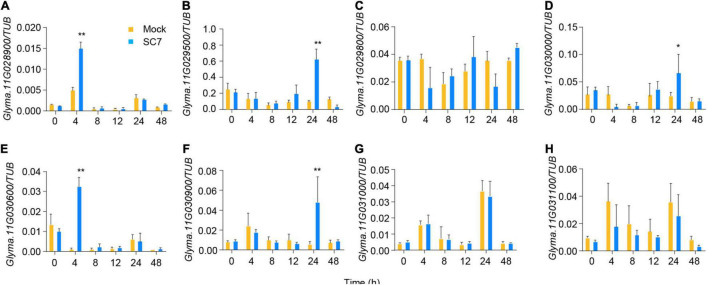
Expression analysis of eight candidate genes in leaves after SC7 inoculation. Values are mean ± s.e.m. (*n* = 3 biologically independent samples). A Student’s *t*-test was used to generate the *P*-values. *P < 0.05; **P < 0.01. Orange boxes represent mock treatment and blue boxes represent treat with SC7 stain in **(A–H)**, respectively.

We next tested the expression of the eight candidate genes in response to SC7 or mock inoculation of DN50 leaves. Five of eight genes showed altered expression patterns following SC7 treatment as compared to mock inoculation: the expression levels of *Glyma.11G028900* and *Glyma.11G030600* were induced at 4 h post-inoculation; *Glyma.11G029500*, *Glyma.11G030000*, and *Glyma.11G030900* were upregulated at 24 h post-inoculation; however, transcript levels of *Glyma.11G029800*, *Glyma.11G031000*, and *Glyma.11G031100* did not change after SC7 inoculation (Figures 6A–H). These results suggested that *Glyma.11G028900*, *Glyma.11G030600*, *Glyma.11G029500*, *Glyma.11G030000*, and *Glyma.11G030900* were the main candidate genes for the *R_*SMV*_-11* locus. Of these genes, only *Glyma.11G028900* harbored a SNP and InDel variation in its coding sequence, resulting in a frameshift (Figure 2B). In addition, according to gene function annotation in the Phytozome database (Liu et al., 2016), *Glyma.11G028900* corresponds to *GmMATE68*, a gene that encodes a multidrug and toxic compound extrusion (MATE) transporter. MATE transporters result in pleiotropic phenotypes, including enhanced plant disease resistance (Nawrath et al., 2002; Ishihara et al., 2008). Taken together, these results indicate that *GmMATE68* is a likely candidate gene for the *R_*SMV*_-11* locus.

## Discussion

Race-specific resistance or basal resistance are major traits that affect soybean yield (Poland et al., 2009; Yasuda et al., 2015). Therefore, identifying resistance genes or resistance-associated QTLs is important for breeding crops with enhanced disease resistance. SMV is a serious threat to soybean production and seed quality worldwide (Adams et al., 2005); however, only a few QTLs linked with SMV resistance and only two resistance genes, *Rsv4* and *Rsc4*, have been cloned (Ishibashi et al., 2019; Yin et al., 2021). Although forward genetics approaches such as positional cloning can successfully isolate candidate genes for diverse traits, they require considerable labor and time (Salvi and Tuberosa, 2005). As an alternative, the next-generation sequencing technology of BSA-seq can quickly detect QTLs for important agronomic traits over wide ranges of experimental variables. In this study, we employed BSA-seq to identify a new candidate QTL on soybean chromosome 11, *R*_*SMV*_-*11*. Our findings also confirmed that *R*_*SMV*_-*11* confers basal resistance to SMV. Overall, our data provide valuable information regarding the genetic basis of SMV resistance in soybean.

Many putative genes have been identified in soybean by fine-mapping and genetic diversity (Lu et al., 2017; Li et al., 2018; Ishibashi et al., 2019; Dong et al., 2021). For example, *J*, a major classical locus conferring the long-juvenile trait, was cloned within a 239-kb region between markers M1 and M3. Only one gene, *EARLY FLOWERING 3* (*ELF3*), differed in sequence between the parental lines in this region (Lu et al., 2017). Furthermore, the broad-spectrum SMV resistance gene *Rsv4* was cloned by fine-mapping, and encodes an RNase H family protein with dsRNA-degrading activity (Ishibashi et al., 2019). In the present study, the soybean cultivar DN50 clearly showed improved SC7 resistance compared to XQD (Figure 1A). To clone the key gene associated with SC7 resistance, we produced an F_2_ population by crossing DN50 and XQD (Figure 2A). The F_6_ population was generated by self-fertilization, and analysis of this population localized *R*_*SMV*_-*11* to a 207-kb region between markers M24 and M28 (Figure 3A), a region harboring 25 genes (Figure 4A). We thus identified a novel locus, *R*_*SMV*_-*11*, conferring resistance to SC7.

Multidrug and toxic compound extrusion (MATE) family members are widely distributed in bacteria, fungi, mammals, and plants (Omote et al., 2006). MATE transporters are involved in a wide range of biological processes in plants, such as the transport of secondary metabolites (Shoji et al., 2009; Zhao and Dixon, 2009), detoxification of toxic compounds or metals (Li et al., 2002; Zhou et al., 2019), and regulation of disease resistance (Nawrath et al., 2002; Ishihara et al., 2008). For example, GmMATE75, GmMATE79, and GmMATE87 are plasma membrane–localized MATE family members whose overexpression resulted in aluminum-induced citrate efflux in soybean (Zhou et al., 2019). Moreover, *EDS5*, which is homologous to members of the MATE family, responds to salicylic acid–dependent signaling, and its overexpression leads to enhanced viral resistance in *Arabidopsis* (Nawrath et al., 2002; Ishihara et al., 2008). In this study, we analyzed the genetic variation, tissue-specific expression pattern, and SC7-induced expression of candidate genes located within the 207-kb region harboring *R*_*SMV*_-*11*. According to gene function annotation in the Phytozome database, *Glyma.11G028900* (*GmMATE68*) encodes a MATE transporter (Liu et al., 2016). There were 22 SNPs and four InDel mutations detected in the *GmMATE68* sequence of XQD, resulting in a protein frameshift (Figure 4B). Our data also demonstrated that *GmMATE68* is expressed in all tissues and is induced following SC7 treatment (Figures 5A, 6A). Our results along with those from the literature suggest that *GmMATE68* may be a strong candidate gene for *R*_*SMV*_-*11*.

Overall, our research provides an important strategy for the rapid identification of target genes controlling key agronomic traits, and our results will promote the development of functional genomics in crops.

## Data Availability Statement

The datasets presented in this study can be found in online repositories. The names of the repository/repositories and accession number(s) can be found in the article/Supplementary Material.

## Author Contributions

QC and ZC designed the experiments and wrote the article. YZ, JS, MY, HD, YL, LD, and KH performed the research. LW, XS, WL, XY, HX, QD, MZ, SL, and YL analyzed the data. All authors contributed to the article and approved the submitted version.

## Conflict of Interest

The authors declare that the research was conducted in the absence of any commercial or financial relationships that could be construed as a potential conflict of interest.

## Publisher’s Note

All claims expressed in this article are solely those of the authors and do not necessarily represent those of their affiliated organizations, or those of the publisher, the editors and the reviewers. Any product that may be evaluated in this article, or claim that may be made by its manufacturer, is not guaranteed or endorsed by the publisher.
