# Supplementary material for: Identifying Quantitative Trait Loci and Candidate Genes Conferring Resistance to Soybean Mosaic Virus SC7 by Quantitative Trait Loci-Sequencing in Soybean
Source: Front Plant Sci. 2022 Feb 28;13:843633. doi: 10.3389/fpls.2022.843633 (PMC8919070; doi:10.3389/fpls.2022.843633)
Supplement: Supplementary file 1 [file Table_1.DOC]

Supplementary Table 1 Primers used for fine-mapping and qRT-PCR in this study.

Primers for fine-mapping

| Primers name | Primers sequences (5’-3’) |
| --- | --- |
| M1-F | TCTCGCATCCAATCACAT |
| M1-R | CAGCCCAATACTATTTGTTTA |
| M24-F | TGCGTGAGTGAATAAATG |
| M24-R | CTAGAAAATGTTTAAATTATGT |
| MRP1-F | CCAAAAATGGGCTTCCGAT（Nde I） |
| MRP1-R | GGTGGGAAGAGTTATGGTTTGAG（Nde I） |
| M25-F | CCATAAAAAGAATACAAGAGCC |
| M25-R | ATCCACCATGATTTCAAACC |
| M26-F | CGCAACCACTTCCCGATTT |
| M26-R  M27-F  M27-R  M28-F  M28-R  M32-F  M32-R  M35-F  M35-R  M37-F  M37-R  11-56-F  11-56-R | CATTGTAGGGCGAGAAGATTGA  AAGCCTCTTATGTGTAAAAAAA  TCATTGTGATTCAGACGATTAC  GCAATCTACTCGGCGTTTC  GGTTTCTACTTTTGTGATGGTGT  GATGGTGCCTGAACAAAT  ATGGGACTGCTATGTTGC  CATACAGCACGGGAGAAA  AGGACCATACATACACGAGAC  GGCTCAACCCTCCAACTC  AATACAAGTGTGATCACCTACAA  ATCCAATATCACCATCACCAC  AGTGACCTTCCGCTGTCG |

Primers for qRT-PCR

| Primers name | Primers sequences (5’-3’) |
| --- | --- |
| qGlyma.11G028900-F | CAAGACAGCCATCACAACAGC |
| qGlyma.11G028900-R | AAGGAGTCTGAAGTTTTTGGCA |
| qGlyma.11G029500-F | AGGACCATCAGGGCGAAGA |
| qGlyma.11G029500-R | TCCAGGGAATAACCTTTCAACC |
| qGlyma.11G029800-F | CCTCAAGTCCCGTTCGGTTAT |
| qGlyma.11G029800-R | TGGGCGTGGGCAAGTTTT |
| qGlyma.11G030000-F | GCAACGACCACCGCAAAG |
| qGlyma.11G030000-R | TTTGAAATGTGTGGGGGTATCTC |
| qGlyma.11G030600-F | ACTCAGGAAGAAGGCTGGGTT |
| qGlyma.11G030600-R | TGAAAGGGTAGTGCTGGTGGTA |
| qGlyma.11G030900-F | TTTGCCTCCACTGCTTGCC |
| qGlyma.11G030900-R | CTGAGCATCACCAAGGCGTA |
| qGlyma.11G031000-F | GGCGGCTTGAGTTGGTTTC |
| qGlyma.11G031000-R | GGGCTTCGCTTACTTTGATTTG |
| qGlyma.11G031100-F | TCGCTCCGAACGAATCTGTC |
| qGlyma.11G031100-R  *SC7-CP-*F  *SC7-CP-*R | CGATTTCAAATTCAGCAGCG  CAGATGGGCGTGGTTATGA  ACAATGGGTTTCAGCGGATA |
